# Antioxidant effect of Kimchi supplemented with Jeju citrus concentrate and its antiobesity effect on 3T3‐L1 adipocytes

**DOI:** 10.1002/fsn3.1138

**Published:** 2019-07-15

**Authors:** Ye‐Rang Yun, Sung-Hee Park, In‐Hwan Kim

**Affiliations:** ^1^ Industrial Technology Research Group, Research and Development Division World Institute of Kimchi Gwangju Korea; ^2^ Jeju in Jeju Farm Corporation Seogwipo‐si Korea

**Keywords:** 3T3‐L1 adipocytes, antiobesity, antioxidant, citrus concentrate, health effects of citrus, Kimchi

## Abstract

Citrus is cultivated throughout Jeju Island and is thought to possess some medicinal properties. Citrus concentrate is the most extensively utilized form of citrus in the food industry. In this study, antioxidant and antiobesity effects of Kimchi supplemented with citrus concentrate were investigated. Prepared Kimchi was infused with 7% citrus concentrate (CK) and freeze‐dried for analysis. Normal Kimchi (NK) without citrus concentrate was utilized as a control. Total phenol content (TPC), total flavonoid content (TFC), and antioxidant activities were examined. Cytotoxicity, intracellular triglycerides (TG), and total cholesterol (TC) levels in 3T3‐L1 adipocytes were also measured. Additionally, the inhibitory effects on lipid accumulation were trialed by measuring the oil‐red O (ORO)‐stained cells. TPC, TFC, and antioxidant activities of CK were significantly higher than those of NK (*p* < .05). CK showed less cytotoxicity and attenuated the lipid accumulation at all concentrations by reducing TG and TC levels compared to NK. The inhibitory effect of CK on lipid accumulation was observed via reducing ORO‐stained lipid droplets. Consequently, the antioxidant and antiobesity effects of CK were revealed in vitro. Furthermore, the addition of citrus may provide competitive price due to low production costs as well as health functionality.

## 1 INTRODUCTION

Citrus is a genus of multiple flowering bushes and assorted trees which produce fruit from the family *Rutaceae* including oranges, lemons, lines, and grapefruits (Meiggs, 1982). Jeju Island is a lush volcanic island with a temperate and humid climate located in South Korea with mild winters perfect for growing citrus. Citrus fruits grown on this island are touted as having beneficial health effects with high amounts of vitamin C, B6, and flavonoids (Tundis et al., 2012; Tundis, Loizzo, & Menichini, 2014). Tanizawa et al. (1992) investigated the antioxidant activity of citrus species and reported that antioxidant activity of immature fruit was greater than that of mature fruit. These beneficial effects were commonly observed in the citrus peel. Bioconversion of the glycoside form of *Citrus unshiu* peel extract inhibited adipogenic activity by suppressing insulin‐induced protein expression and increasing adipolytic activity (Lim et al., 2015). Another study monitored the intake of *Citrus unshiu* peel pellet for 4 weeks and found that it was effective in weight control and decreased lipid profiles of subjects such as triglycerides (TG), total cholesterol (TC), and low‐density lipoprotein cholesterol (LDL‐C) level (Kang, Song, Lee, Chang, & Lee, 2018).

As a citrus product, citrus concentrate is widely utilized and applied in the food industry. Some functional health effects relating to ingestion of citrus concentrate have previously been investigated. For instance, Oikeh et al. reported that four citrus juice concentrates *Citrus aurantifolia, limon, paradise,* and *tangerina* showed antimicrobial effects as well as antioxidant effects (Oikeh, Omoregie, Oviasogie, & Oriakhi, 2015). In addition, Cho, (2009) found that supplementation with mandarin concentrate showed antiobesity effects in rats with diet‐induced obesity, by decreasing body adipose tissue, weight, and lipid levels. Moreover, citrus concentrate has been widely utilized in a variety of ways to develop new food processing products. For instance, the addition of 6% citrus concentration was found to increase amino nitrogen and reduce sugar content, leading to improvements in the quality of Gochujang (Chae et al., 2008). Another study found that the most suitable citrus concentration for nutritional cereal bars was 15%, as it was rated highest in the sensory evaluations (Park, Oh, & Cha, 2014; Park, Choi, Ko, Rha, & Lee, 2014). Thus, the addition of citrus concentrate has the potential to improve both the quality and function of food.

Kimchi has been extensively studied and reported, as it is also thought to have medicinal properties. Kimchi lactic acid bacteria likely contribute to this medicinal activity by providing beneficial bacteria to the gut. Within Korea, consumption of Kimchi is high since it is a popular Korean fermented food. A clinical trial by Han et al. (2015) found that consumption of fermented Kimchi influenced metabolism‐related gene expression as well as gut microbial composition. Similarly, two lactic acid bacteria from Kimchi (*Lactobacillus plantarum* strains, DSR M2 and DSR920) showed amelioration of obesity in diet‐induced obese mice (Lee et al., 2018). In addition, Vasilis et al. recently reported that life expectancy of the South Korean people will be at its the highest level, 86.7 years, in 2030 (Kontis et al., 2017). Further, Shea noted that this higher life expectancy is in part due to Korea's traditional diet consisting largely of Kimchee (Kimchi) (Shea, 2017). However, although the international interest in Kimchi is increasing, domestic consumption of Korean Kimchi is gradually decreasing due to the entry of cheap Chinese Kimchi into the domestic Kimchi market. Thus, the development of differentiated Kimchi is necessary in order to compensate for this.

This study investigated the antioxidant and antiobesity effects of Kimchi supplemented with citrus concentrate (CK). Initially, Kimchi with or without 7% citrus concentrate was freeze‐dried for analysis. Normal Kimchi (NK) without citrus concentrate was utilized as a control. Total phenol content (TPC) and total flavonoid content (TFC) of CK were examined. Antioxidant effects of CK were investigated by total antioxidant capacity (TAC) and ferric reducing antioxidant power (FRAP) assay. The antiobesity effect of CK was investigated by measuring the intracellular TG and TC levels of 3T3‐L1 adipocytes. In addition, the inhibitory effect of CK upon lipid accumulation was measured via oil‐red O (ORO) staining.

## 2 MATERIALS AND METHODS

### 2.1 Chemicals

Citrus concentrate utilized in this study was purchased from Jeju Island (ES Food, Kyonggi, Korea). Gallic acid, quercetin, ORO solution, 3‐isobutyl‐1‐methylxanthine (IBMX), dexamethasone, insulin, and TAC kit of Sigma Aldrich were purchased. FRAP kits of Ann Arbor were purchased (Ann Arbor, MI, USA). Cell counting kit‐8 (CCK‐8) of Dojindo was purchased (CCK‐8, Dojindo, Japan).

### 2.2 Preparation of Kimchi with 7% citrus concentrate

**Figure 1:**
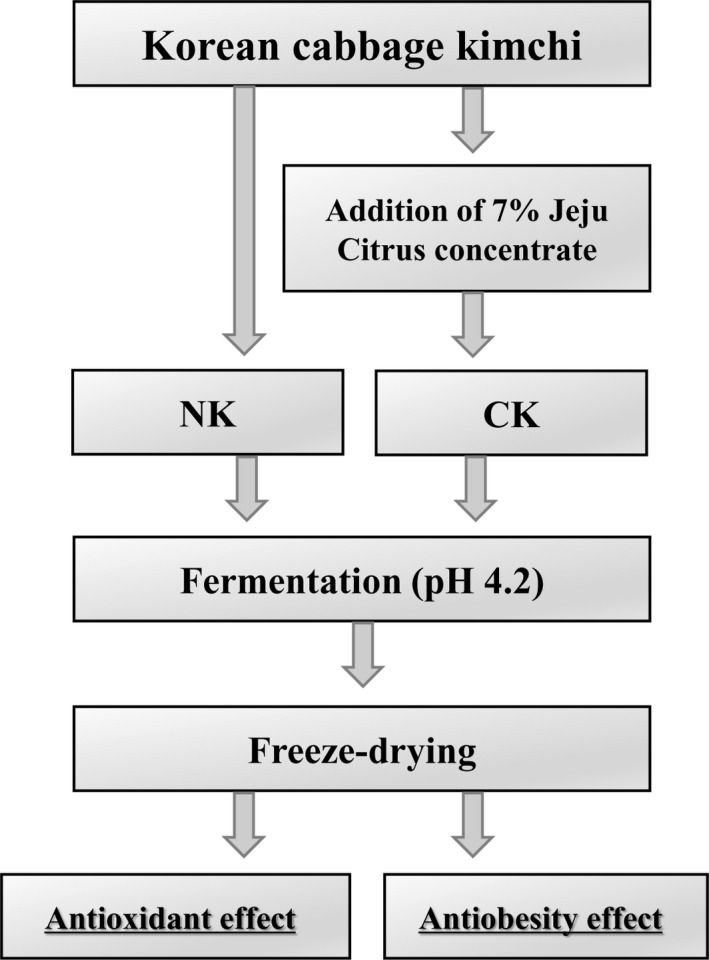
The preparation of Kimchi supplemented with citrus concentrate. Normal Kimchi (NK) was made with brined Korean cabbage, red pepper, garlic, ginger, onion, and radish, while Kimchi added with citrus concentrate (CK) was made with 7% of citrus concentrate instead of sugar sources including stevia, corn syrup, and onion. NK and CK were fermented until pH 4.2 and freeze‐dried

Normal Kimchi (NK) was made with brined Kimchi cabbage and Kimchi seasoning (3:1). As a Kimchi seasoning, red pepper, radish, onion, glutinous rice, garlic, ginger, shrimp sauce, anchovy sauce, stevia, and corn syrup were used. To make CK, 7% citrus concentrate was added instead of sugar sources, including stevia, corn syrup, and onion. In addition, red pepper was reduced by 80% compared with NK. NK and CK were fermented until they reached a pH of 4.2 and then freeze‐dried for analysis. Freeze‐dried NK and CK were utilized for the following experiments. The preparation of CK was presented in Figure 1. In the screening test, Jeju citrus concentrate was 60 brix and hesperidin content was 6807.34 mg/kg. Hesperidin content of CK (containing 7% citrus concentrate) was 1097.83 mg/kg, and NK was very low content. Based on these results, antiobesity effect of hesperidin in CK can be expected.

### 2.3 Total phenol content measurement

Measurement of total phenol content (TPC) was conducted according to Folin–ciocalteau's method (Durazzo, Turfani, Azzini, Maiani, & Carcea, 2013). The optical density (OD) value of TPC was measured at 700 nm in a microplate reader. TPC was expressed in mg of gallic acid per equivalent gram of extract (mg GAE/g) based on a linear equation for a standard curve prepared with gallic acid.

### 2.4 Total flavonoid content measurement

Measurement of TFC was conducted utilizing Moreno's method (Chang, Yang, Wen, & Cherrn, 2002). The OD value of FPC was measured at 415 nm. TFC is expressed in mg of quercetin equivalent per gram of extract (mg QE/g), based on a linear equation for standard curve prepared with quercetin.

### 2.5 Total antioxidant capacity (TAC) assay

Antioxidant assay was conducted via use of a TAC kit that measured the resulting TAC value. The OD value of TAC was measured at 570 nm. TAC value was expressed in μM_,_ based on a linear equation for Trolox standard curve.

### 2.6 Ferric reducing antioxidant power (FRAP) assay

FRAP value was measured by using FRAP^™^ colorimetric detection kit. The OD value of FRAP was measured at 560 nm. FRAP value is expressed as μM, based on a linear equation for Fe (II) standard curve.

### 2.7 Cell culture

3T3‐L1 preadipocytes derived from mouse were obtained from the American Type Culture Collection (ATCC) and grown in DMEM containing 10% fetal calf serum (FCS) and 1% penicillin/streptomycin until confluence. Then, 3T3‐L1 preadipocytes were differentiated in DMEM with 10% fetal bovine serum (FBS), 0.5 mM IBMX, 1 μM dexamethasone, and 5 μg/ml insulin (MD1). After 2 days, 3T3‐L1 adipocytes were maintained in DMEM with 10% FBS and 5 μg/ml of insulin (MD2) until 8 days.

### 2.8 Cytotoxicity analysis

Cytotoxicity of 3T3‐L1 preadipocytes was measured by CCK‐8 kit. 3T3‐L1 preadipocytes were placed with 1 × 10^4^ cells/well and incubated with NK or CK (0, 0.05, 0.25, 0.5, 1, and 2.5 mg/ml) for 1 day. Thereafter, cells were washed with phosphate‐buffered saline (PBS) three times. Then, cells were reacted with 20 µl of CCK‐8 solution for 4 hr. Absorbance was read at 450 nm.

### 2.9 Intracellular triglyceride and total cholesterol level

To measure intracellular TG and TC levels, the lipids of differentiated 3T3‐L1 adipocytes were initially extracted in 700 μl of solvent mixture (chloroform/methanol/H_2_O mixture, 8:4:3, v/v/v). After incubation at room temperature (RT) for 60 min, the bottom organic layer was obtained with centrifugation at 800 *g* for 10 min and dried. Then, it was dissolved in 30 μl of ethanol and examined for TG and TC level using an enzyme reaction kit (Asan pharmaceutical).

### 2.10 Oil‐red O staining

The inhibitory effect of CK on lipid accumulation was observed by measuring and quantifying ORO from stained 3T3‐L1 adipocytes. Differentiated cells were washed and fixed in 10% formalin for 30 min. After washing, cells were stained with an ORO solution for 15 min at RT. Then, stained cells were washed and captured under a light microscope. Next, ORO from stained 3T3‐L1 adipocytes was extracted with 4% NP‐40 to quantify and the absorbance was read at 510 nm.

### 2.11 Statistical analysis

Data were expressed as the mean ± *SD*. Significant difference was analyzed by paired two‐tailed Student's *t* test using GraphPad Prism 7, respectively (GraphPad Software Inc., San Diego, California, USA). Statistical significance was accepted in *p* values of <.05.

## 3 RESULTS AND DISCUSSION

### 3.1 Total phenol and total flavonoid contents of Kimchi with citrus concentrate

**Figure 2:**
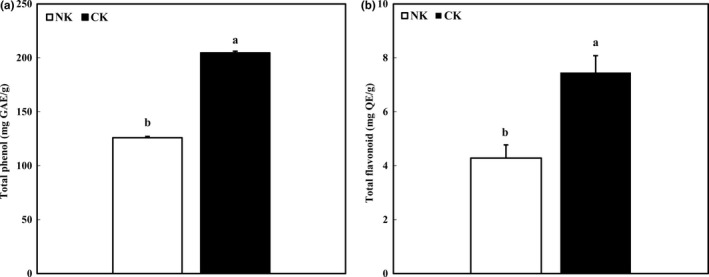
Total phenol and flavonoid level of Kimchi added with citrus concentrate. (a) Total phenol content and (b) total flavonoid content. Data were expressed as mean ± *SD*. Different letters indicate significant difference between NK and CK (*p* < .05)

Total phenol content and TFC were measured before verifying antioxidant activities of CK. Figure 2 depicts the TPC and TFC of NK and CK. TPC of CK was approximately 62% higher than that of NK. Woo, Woo, and Jeong (2006) reported that TPC of commercial Kimchi (C ~ F) showed around 120 mg% and TPC of Kimchi adding germinated brown rice extract was increased by 156.87 mg%. Similarly, Ha et al. found that TPC of Kimchi with added mustard leaf, mushroom, and sea tangle (functional Kimchi) was also increased compared with that of the control (Ha & Kang, 2018). TPC and antioxidant activity show a positive correlation. For example, water extracts of 20 medicinal plants showed higher antioxidant activity as TPC was increased (Kim, Baik, Kim, Kim, & Ryu, 2004). According to Wang's results, phenolic compounds dominantly contributed to the antioxidant activity of citrus extracts (Wang et al., 2017). TFC of CK was also higher than that of NK at 7.46 mg/ml (*p* < .05). Based on these results, the antioxidant effect of CK could be expected.

### 3.2 Total antioxidant capacity of Kimchi with citrus concentrate

**Figure 3:**
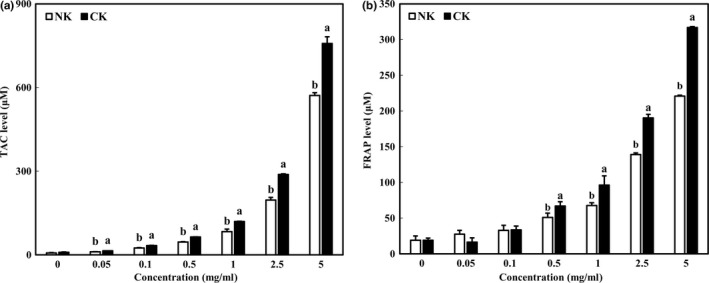
Antioxidant activities of Kimchi supplemented with citrus concentrate. (a) Total antioxidant capacity and (b) ferric reducing antioxidant power. Data were expressed as mean ± *SD*. Different letters indicate significant difference between NK and CK (*p* < .05)

Antioxidant activities were estimated with TAC and FRAP assays. As shown In Figure 3a, TAC values were dose‐dependently increased in both NK and CK. In addition, the TAC value of CK was significantly higher than that of NK in all concentrations. Over 1 mg/ml concentration, the TAC value of CK was higher than 100 μM. Particularly, TAC value of CK at 5 mg/ml was 758.10 μM and was increased about 32% compared with that of NK. Similar to the results of this study, some studies have also reported that the addition of various substances increased antioxidant activity of Kimchi. Lee et al. reported that Kimchi seasoning with black garlic increased antioxidant activity via increasing the 2,2‐diphenyl‐1‐picrylhydrazyl (DPPH) electron donating activity (Lee & Yoon, 2017). Functional Kimchi also increased DPPH radical scavenging, 2,2'‐azino‐bis(3‐ethylbenzothiazoline6‐sulfonic acid, ABTS) anion scavenging, and oxygen radical absorbance capacity (ORAC) value (Ha & Kang, 2018). In another study, the higher TPC and TFC of CK showed the higher antioxidant activity (Zhang et al., 2014). These results indicated that the addition of citrus concentrate increased the antioxidant activity of Kimchi.

### 3.3 Ferric reducing antioxidant power of Kimchi with citrus concentrate

As consistent with TAC results, FRAP values of CK were significantly higher than those of NK at all concentrations (Figure 3b, *p* < .05). At 5 mg/ml, the FRAP value of CK was about 42% higher than that of NK. Similarly, Ha et al. reported that functional Kimchi showed a higher FRAP value by 1.6 compared with that of the control (Ha & Kang, 2018). In Wang's study, citrus fruit containing more phenolics showed higher antioxidant activity (Wang et al., 2017). Taken together, the antioxidant effect of CK was confirmed in comparison with NK. Similar to TPC and TFC results, the addition of citrus concentrate may improve the antioxidant effect of Kimchi.

### 3.4 Cytotoxicity of Kimchi with citrus concentrate on 3T3‐L1 preadipocytes

**Figure 4:**
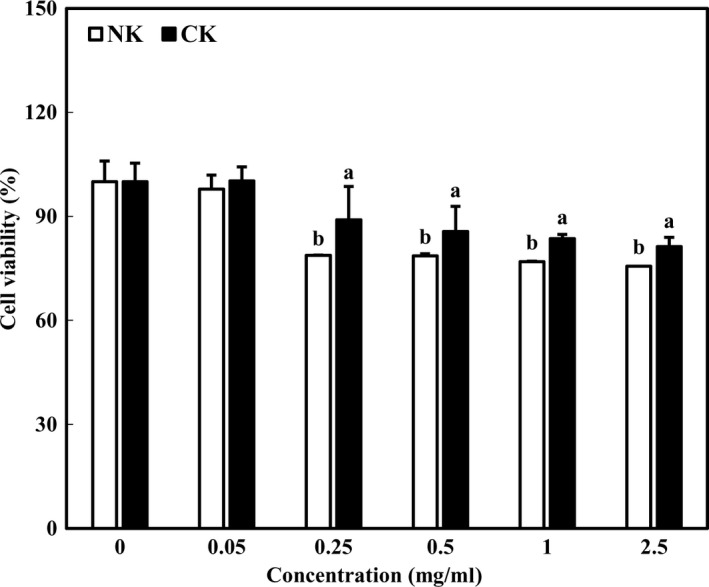
Cytotoxicity of Kimchi added with citrus concentrate on 3T3‐L1 preadipocytes. Data were expressed as mean ± *SD*. Different letters indicate significant difference between NK and CK (*p* < .05)

Cytotoxicity of Kimchi supplemented with citrus concentrate on 3T3‐L1 preadipocytes was examined using a CCK‐8 assay. Figure 4 showed the cytotoxicity of NK and CK in various concentrations (0, 0.05, 0.25, 0.5, 1, and 2.5 mg/ml). At concentrations of 0.05–0.5 mg/ml, the cell viability of CK was over 80%, indicating a low cytotoxicity. Unexpectedly, cytotoxicity of CK increased at concentrations above 1 mg/ml. This cytotoxicity of CK may be due to different pretreatment and different cell line. In this study, freeze‐dried Kimchi without special extraction was used for this experiment, leading to being cytotoxic. In addition, cytotoxicity also depends on the concentration of the sample but also on the type of cell. For instance, cytotoxicity of Kimchi extract was different in keratinocytes and fibroblasts (2.5–10 and 10%–20%, respectively) (Ryu et al., 1997). Therefore, low cytotoxicity of NK and CK may be expected in other cell lines. In comparison cytotoxicity between CK and NK, cytotoxicity of NK was higher than that of CK. The lower cytotoxicity of CK may be due to various physiologically active substances in citrus concentrate. Considering cytotoxicity, the usage of 0.5 mg/ml concentration would be appropriate for the application.

### 3.5 Intracellular triglycerides and total cholesterol levels of Kimchi with supplemented citrus concentrate on 3T3‐L1 adipocytes

**Figure 5:**
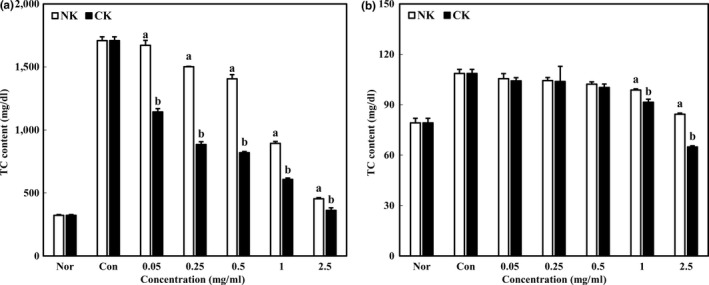
Intracellular triglyceride and total cholesterol level of Kimchi with additional citrus concentrate on 3T3‐L1 adipocytes. Data were expressed as mean ± *SD*. Different letters indicate significant difference between NK and CK (*p* < .05)

To reveal the inhibitory effects of CK on lipid accumulation of 3T3‐L1 adipocytes, intracellular TG and TC levels were measured. Intracellular TG levels of NK and CK were dramatically decreased in a dose‐dependent manner, leading to suppression of adipocyte differentiation (Figure 5a). Particularly, TG levels of NK and CK at 2.5 mg/ml were 454.03 and 362.48 mg/dl, respectively. In comparison with control, TG levels of NK and CK were decreased by about 74 and 79%, respectively. The antiobesity effect of Kimchi in 3T3‐L1 adipocytes has not been reported; however, studies on Kimchi‐derived lactic acid bacteria were commonly reported (Lee et al., 2015; Moon et al., 2012; Park, Oh, et al., 2014; Park, Choi, et al., 2014). In recent days, Park, Lee, and Park (2018) reported that Kimchi prepared with solar salt showed an antiobesity effect by decreasing the lipid accumulation of 3T3‐L1 adipocytes.

Intracellular TC levels were also measured. Figure 5b shows that TC level was decreased in both NK and CK compared with that in the control. TC levels of NK and CK at 2.5 mg/ml were 84.35 and 54.85 mg/dl, respectively. In similar with TG results, CK significantly decreased intracellular TC level. NK also showed the inhibitory effects on lipid accumulation of 3T3‐L1 adipocytes, but showed an outstanding inhibitory effect of CK supplemented with citrus concentrate. These results indicated that citrus concentrate of CK has a potential to inhibit the lipid accumulation. In Sato's research, immature citrus fruit power intake showed the antiobesity effect by decreasing blood lipids (Sato, Goto, Inoue, Miyaguchi, & Toyoda, 2019).

### 3.6 Inhibitory effect of Kimchi with citrus concentrate on lipid accumulation

**Figure 6:**
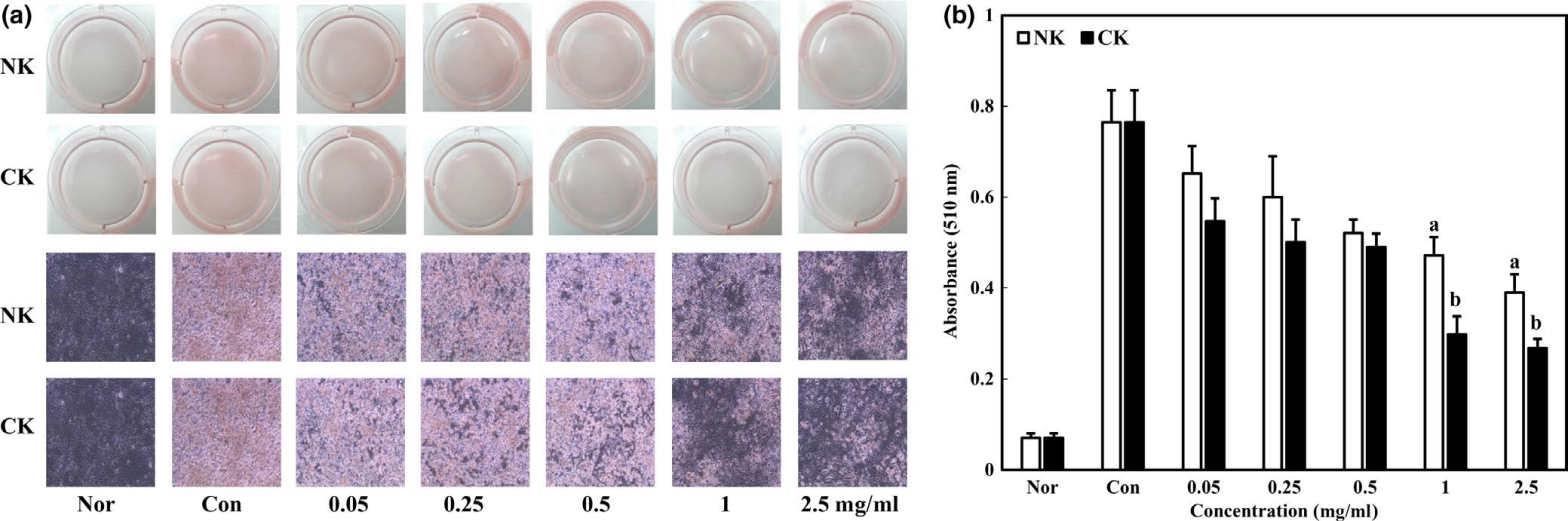
Inhibitory effects of Kimchi added with citrus concentrate on lipid accumulation in 3T3‐L1 adipocytes. (a) ORO‐stained image and (b) quantification of ORO from stained 3T3‐L1 adipocytes. Data were expressed as mean ± *SD*. Different letters indicate significant difference between NK and CK (*p* < .05)

The inhibitory effect of NK and CK on lipid accumulation was evaluated by ORO staining. ORO staining is widely used for the detection of lipids in cells and tissues. The increase in adipocytes is known to be closely related to the accumulation of lipid content (Poudel et al., 2015). Therefore, ORO‐stained cells indicate the degree of lipid accumulation in 3T3‐L1 adipocytes. As shown in Figure 6, ORO‐stained cells of NK and CK were dose‐dependently and significantly decreased (*p* < .05). In quantification results, the stained lipid droplet level of CK was lower than that of NK. The absorbance of NK and CK was 0.39 and 0.27, respectively. Similarly, bioconversion of *Citrus unshiu* with cytolase (CU‐C) showed significant inhibitory effect on lipid accumulation at 0.5mg/ml (Lim et al., 2015). These results indicated that citrus concentrate inhibited lipid accumulation in 3T3‐L1 adipocytes, suggesting its antiobesity effect.

## 4 CONCLUSION

In this study, CK showed higher TPC and TFC than NK. Similarly, antioxidant activities of CK were confirmed through TAC and FRAP value results compared with those of NK. Antiobesity effects of CK were exhibited by decreasing TG and TC levels of 3T3‐L1 adipocytes, showing low cytotoxicity. The antiobesity effect of CK was confirmed by inhibiting lipid accumulation in ORO staining results. Taken together, CK showed the most powerful antioxidant and antiobesity effect. Based on these results, the antiobesity effect of CK should be verified in animal trials in future.

## CONFLICT OF INTEREST

There are no conflicts of interest.

## ETHICAL STATEMENT

This study does not involve any human testing.
